# Moroccan *Lycium intricatum* Berries: Therapeutic Insights, Phytochemical Profile, and Biological Activities

**DOI:** 10.1155/ijfo/8532330

**Published:** 2026-04-24

**Authors:** Khawla Bouaouda, Mourad Oukheda, Hanane Choubbane, Chaimaa Elagdi, Anass Kettani, Rachid Saile, Hassan Taki

**Affiliations:** ^1^ Department of Biology, Faculty of Sciences Ben M′Scik, Laboratory of Biology and Health, University Hassan II, Casablanca, Morocco, uh2c.ac.ma; ^2^ Department of Chemistry, Faculty of Sciences and Technology of Gueliz, Laboratory of Sustainable Development and Health, University Cadi Ayyad, Marrakech, Morocco, uca.ma; ^3^ Department of Biology, Faculty of Sciences Ben M′Scik, Laboratory of Ecology and Environment, University Hassan II, Casablanca, Morocco, uh2c.ac.ma

**Keywords:** antioxidant activity, antiprotein denaturation activity, extraction method, extraction solvent, HPLC-UV-MS/MS, *Lycium intricatum* berries

## Abstract

This study focuses on examining the chemical composition of *Lycium intricatum* berries, a plant historically used by the Moroccan population but not extensively studied. Using HPLC‐UV‐MS/MS analysis, we identified significant levels of bioactive compounds. Quercetin and its derivatives were found to be the most abundant compounds across all extracts. The fruit was also found to contain two major carotenoids, 8‐Apo‐b‐carotenal and alloxanthin, and notable levels of phenolic and chlorogenic acids, well‐regarded for their therapeutic effectiveness in treating eye conditions, substantiating the fruit′s historical use. Quantification of phenolic compounds revealed a high content of total polyphenols, total flavonoids, and condensed tannins in all extracts, with solvent‐water mixtures proving to be the most effective for extraction. In terms of biological activities, the antioxidant potential was evaluated using DPPH and hydrogen peroxide tests, showing the ethanol: water (50:50) extract to exhibit strong antioxidant activity (IC_50_ = 273.90 ± 0.96 *μg*/mL, IC_50_ = 30.96 ± 1.95 *μg*/mL), respectively. Furthermore, the acetone: water (80:20) extract demonstrated the most potent antiprotein denaturation effect, assessed using BSA and egg albumin tests. A comparison of extraction methods highlighted that ultrasound‐assisted extraction was more efficient and cost‐effective compared to traditional maceration, especially when using solvents such as methanol, ethanol, acetone, and water. Interestingly, ethyl acetate and hexane were found to facilitate superior extraction through maceration, providing valuable insights into effective extraction strategies.

## 1. Introduction

Throughout human history, plants have historically been the main source of medication, serving as the foundation for numerous traditional medical procedures in various cultures [1, 2]. Traditional medicine has long been a crucial component of the healthcare system in Morocco, a nation with a rich and diverse flora [3]. The prevalence of conventional treatments, frequently made from local plant species, is a reflection of how deeply rooted traditional medicine is in Moroccan culture and history. The Moroccan population has traditionally used medicinal and aromatic plants as therapeutic agents to heal a multitude of diseases [3, 4].

Several local plant species have been incorporated into traditional medicinal preparations, including infusions and other traditional forms of medication, for the treatment of various diseases. The part of plants commonly used encompasses the fruit, leaf, and root [5].

*Lycium* is a genus of approximately 80 species distributed all over the world, and is disjunctly in temperate to subtropical regions in South America, southern Africa and Asia. [6].

The three most common species in Morocco are *Lycium intricatum* Boiss., *Lycium barbarum* (Munby) Batt., and *Lycium europaeum* L. *L. intricatum or* “awsaj” as also called is a shurby plant, a common fleshy‐fruited, up to 2 m in height. It produces bright orange berry‐shaped fruit when ripe [3, 7]. In addition to Morocco, *L. intricatum* is found in a number of other nations as Portugal, Mauritania, Algeria, Egypt, Saudi Arabia, Tunisia, and Italy [8]. It is among those plant species historically used by the population, and especially the wood for the treatment of women′s sterility [9], Herbalists have traditionally used seeds to treat helminthiasis and stomach problems, and the fruit of *L. intricatum* is believed to have potential benefits in the treatment of eye problems because of its phenolic compounds [8, 10]. It is a species rich in phytochemicals and slightly influenced by commercial breeding. Several compounds were already identified by previous studies carried out in Tunisia and Algeria, notably phenolic acid, flavonoids, fatty acids, phytosterols, vitamin D, as well as a new ionone derivative from *L. intricatum* leaves [11–13]. Despite the plant′s many benefits, there exists only few information, emphasizing the need for extensive research to elucidate its potential uses.

The application of goji berry (the fruit of *L. barbarum*) nutrients and resources has received more attention in recent years due to research and the growing demand for health foods [14, 15]. However, the investigations regarding *L. intricatum* berries remain very limited. This study represents the initial research to explore the phytochemical profile of *L. intricatum* fruit in Morocco.

This work is a continuation of an investigation into underutilized plants in Morocco that were historically used as therapeutic sources [16]. The present work is aimed at identifying bioactive compounds by HPLC‐UV‐MS/MS of different extracts and evaluate the protein antidenaturation effect of *L. intricatum* berry, to investigate the antioxidant capacity and to assess the impact of the extraction method (probe ultrasound assisted extraction [PUAE] and maceration) and solvent (methanol, ethanol, acetone, hexane, ethyl acetate, and water) on these biological properties.

## 2. Materials and Methods

### 2.1. Materials and Equipment

The main equipment used in this study included the following:

• Ultrasound Probe Sonicator: Vibra‐Cell VCX‐130 (20 kHz, programmable, 6 mm probe, United States).
• Centrifuge: Multi‐Rotor Next Generation (Southwest Science, United States) with swing‐out rotor for 6 × 50 mL tubes, speed range 300–4500 rpm, RCF up to 2500 × g.
• Rotary Evaporator: IKA RV 10 digital (IKA‐Werke GmbH & Co. KG, Germany) with universal heating bath, vertical glassware, motorized lift, and digital control.
• UV‐Vis Spectrophotometer: SP‐UV759 (Scitek, China) with automatic cuvette handling.
• HPLC System: liquid chromatography with triple‐quadrupole mass spectrometer (Thermo Fisher Scientific, United State), equipped with a reverse‐phase Kinetex C18 column (100 × 4.6 mm, 2.6 *μ*m, Phenomenex, United States).

### 2.2. Chemicals and Reagents

All chemicals and solvents used in this study were of analytical grade. Methanol, ethanol, acetone, hexane, ethyl acetate, HPLC‐grade methanol, Folin–Ciocalteu reagent, sodium carbonate (Na₂CO₃), gallic acid, sodium nitrite (NaNO₂), aluminum chloride (AlCl₃), sodium hydroxide (NaOH), catechin, vanillin, hydrochloric acid (HCl), hydrogen peroxide (H₂O₂), phosphate buffer, ascorbic acid, bovine serum albumin (BSA), and egg albumin were purchased from Merck (Darmstadt, Germany) and Sigma‐Aldrich (St. Louis, Missouri, United States).

### 2.3. Harvest and Plant Preparation

The species *L. intricatum* is recovered from the Casablanca‐Settat region. The Lambert coordinates are: 33°28 ^′^47 ^″^N 7°56 ^′^49 ^″^W. The plant is allowed to dry away from light and at room temperature before the separation of its organs (leaves, stems, and fruits). The berries are oval to ellipsoidal in shape, generally orange‐red in color, and measure about 5–10 mm in length and 3–6 mm in diameter. The fruit is reduced to powder, using an electric grinder and sieved through a 60‐mesh sieve before stored away from moisture.

### 2.4. Extraction Methods

**Figure 1:**
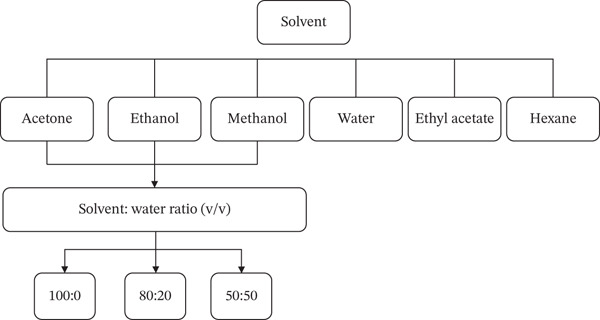
Solvents and mixture of solvents with water used for each extraction method.

A quantity of 2.5 g of plant powder was mixed with 50 mL of distilled water or with different solvents (methanol, ethanol, acetone, hexane, and ethyl acetate) at varying solvent‐water ratios (50:50, 80:20, and 100:0) as shown in Figure 1.

Two extraction methods were used: conventional solid‐liquid extraction (maceration) and PUAE.

The yield of extraction methods is calculated according to the following equation:

(1) Weight of dried crude extract gWeight of dried plant sample g×100

#### 2.4.1. Conventional Solid‐Liquid Extraction (Maceration)

The plant material was mixed with solvent for 24 h under magnetic stirring protected from light. The mixture was then filtered and centrifuged for 10 min at 10,000 rpm. The superior agent was recovered and evaporated to dryness, and then stored for further analysis [16].

#### 2.4.2. Ultrasound Probe‐Assisted Extraction (UPAE)

The powdered plant material was extracted using a probe sonicator. At the highest power levels (400 w), the probe was immersed in each beaker containing plant material and solvent for 15 min. To prevent temperature rise during extraction, an ice bath was used. The extracts were subsequently filtered, centrifuged, and dried using a rotary evaporator. The crude dried extracts were stored until use [16].

### 2.5. Determination of Secondary Metabolites Contents

#### 2.5.1. Total Phenolic Content (TPC)

The Folin–Ciocalteu method was used for the determination of the TPC according to the following protocol [17], 600 *μ*L of distilled water is added to 10 *μ*L of the extract and 50 *μ*L of Folin–Ciocalteu. The mixture is stirred and allowed to stand for 10 min before adding 150 *μ*L of Na_2_Co_3_ (2% *w*/*v*). The volume is supplemented to 1 mL with distilled water and incubated at room temperature for 2 h in darkness. The absorbance was measured at 760 nm.

The results are compared with the gallic acid calibration curve (5–100 *μ*g/mL, *R* ^2^ = 0.998), under the same conditions and expressed as mg gallic acid equivalents (GAE) per g dry weight (DW).

#### 2.5.2. Total Flavonoid Content (TFC)

The determination of total flavonoids was carried out according to the method described previously [18]. Two hundred and fifty microliters of each extract (0.125 mg/mL) was added to 1 mL of distilled water and 75 *μ*L of NaNo2 solution (5%), followed by 75 *μ*L of Alcl3 solution (10%) after 6 min. The mixture was left to stand for another 6 min before adding 1 mL of NaOH (4%) solution. Distilled water was added to bring the final volume to 2.5 mL.

The mixture was left in the dark for 15 min and the absorbance was measured at 510 nm.

Catechin was used as a standard curve (5–400 *μ*g/mL, *R* ^2^ = 0.997) and the results were expressed as mg of catechin equivalents (CE) per g DW.

#### 2.5.3. Condensed Tannin Content (CTC)

A total of 1.5 mL of vanillin (4%) and 750 *μ*L of hydrochloric acid were added to 100 *μ*L of each extract. The mixture was incubated at room temperature for 20 min and the absorbance was recorded at 500 nm. CTC results were counted according to a catechin standard curve (5–400 *μ*g/mL, *R* ^2^ = 0.985), and expressed as mg of catechin equivalent per 100 g of dry weight (mg CE/100 g DW). [19].

### 2.6. In Vitro Antioxidant Activity

#### 2.6.1. 2,2‐Diphenyl‐1‐Picrylhydrazyl Radical Scavenging Assay (DPPH)

The radical scavenging activity was determined following a previously cited method [11]. Five hundred microliters of each extract (dissolved in methanol) in different concentrations was mixed with 1 mL of a DPPH solution (0.1 mM, freshly prepared in methanol). The mixture was stirred well and left to stand in the dark for 60 min. The absorbance was measured at 515 nm and the percent inhibition was calculated using the formula:

(2) %scavenging effect=ADPPH−AsADPPH×100

#### 2.6.2. H_2_O_2_ Scavenging

The H_2_O_2_ scavenging performance was evaluated as follows [20]: 40 mM H_2_O_2_ solution was added to phosphate buffer (50 mM, pH 7.4). All experimental samples (1 mg.mL^−1^) were combined with 0.6 mL of H_2_O_2_ solution, incubated for 10 min, and the absorbance of the mixture was measured spectrophotometrically at 230 nm. Phosphate buffer was used as the blank, and ascorbic acid was used as the standard. Using the following formula, hydrogen peroxide scavenging (%) was calculated.

(3) Hydrogen peroxide scavenging activity=A0−AsA0×100

where Ao is the absorbance of the blank and As is the absorbance in the presence of the ascorbic acid standard or samples.

### 2.7. In Vitro Antiprotein Denaturation Activity

#### 2.7.1. BSA Assay

BSA assay of berry extracts was performed as follow [20]. In brief, 0.45 mL of BSA and 0.05 mL of samples were mixed and incubated for 25 min at 40°C. Phosphate buffer saline (2.5 mL; pH 6.3) was added to the tubes and the absorbance was measured using a spectrophotometer at 660 nm.

The inhibition of BSA denaturation was calculated using the following equation:

(4) %Inhibition of BSA denaturation=A12−AA1×100

where *A*1 is the absorbance of the control, and *A*2 is the absorbance of the sample.

#### 2.7.2. Chicken Egg Albumin (CEA) Assay

The ability of berry extracts to inhibit protein denaturation was investigated using the method described previously [21]. Two milliliter of extract and 0.2 mL of CEA were added to 2.8 mL of PBS solution. Diclofenac was used as standard. All samples were kept at room temperature for 15 min before being heated to 70° C for 10 min. The absorbance was measured at 660 nm and inhibition of egg albumin denaturation was calculated using the following equation:

(5) %Inhibition of egg albumin denaturation=A12−AA1×100

where *A*1 is the absorbance of the control and *A*2 is the absorbance of the test sample.

### 2.8. HPLC‐UV‐MS/MS Analysis

A liquid chromatography system combined with a triple‐quadrupole mass spectrometer (Thermo Fisher Scientific, San Jose, California, United States) was used to qualitatively examine phenolic compounds. The proposed method was tested on a reverse phase Kinetex C18 column (100 × 4.6 mm, particle size 2.6 *μ*m). Gradient separation was carried out according to the method described in [22] using solvent A (0.1% aqueous formic acid) and solvent B (methanol). Phenolic compounds were identified on the basis of retention times, spectra corresponding to nine standards, and the NIST‐MS/MS library.

### 2.9. Statistical Analysis

All of the analyses in this study were done in triplicates. Data were presented as mean ± standard deviation (SD). Statistical analysis was performed using one‐way ANOVA followed by Duncan′s multiple range test. Graphpad Prism 8 for Windows was used to calculate *I* *C*_50_ and Pearson′s correlation coefficients between biological activity assays and the concentrations of plant secondary metabolites (TPC, TFC, and CTC).

## 3. Results

### 3.1. Extraction Yield

The determination of fruit extraction yields by varying the type of solvent and extraction method showed that PUAE is significantly more efficient than maceration (*p* < 0.05), as shown in Table 1. Hexane and ethyl acetate, being nonpolar solvents, showed higher extraction yields during maceration but were less effective in extracting phenolic compounds, particularly under PUAE. The results revealed a significant difference between the extraction yields of the different solvents used. The solvent (ethanol: water, 50:50 *v*/*v*) gave the highest yield for fruit extract followed by (methanol: water, 80:20 *v*/*v*) and (acetone: water, 80:20 *v*/*v*), whereas the lowest yield is obtained with ethyl acetate.

**Table 1:** Extraction yields of different berry extracts of *Lycium intricatum.*

Extraction method	Extraction solvents (solvent:water)		Yield (%)
Maceration	MeOH	100:0	17.84 ± 1.17^cd^
		80:20	34.40 ± 3.56^a^
		50:50	21.02 ± 0.30^bc^
	EtOH	100:0	15.26 ± 2.08^d^
		80:20	20.09 ± 1.66^bc^
		50:50	23.04 ± 0.64^b^
	Acetone	100:0	2.70 ± 0.26^e^
		80:20	20.18 ± 0.89^bc^
		50:50	17.67 ± 0.89^cd^
	H_2_O		18.70 ± 1.17^cd^
	Hexane		1.85 ± 0.41^e^
	Ethyl acetate		1.14 ± 0.37^e^

Probe ultrasound assisted extraction	MeOH	100:0	39.96 ± 0.73^ab^
		80:20	41.10 ± 0.75^ab^
		50:50	29.95 ± 1.39^bc^
	EtOH	100%	17.80 ± 1.59^d^
		80:20	27.15 ± 1.06^c^
		50:50	48.51 ± 8.80^a^
	Acetone	100:0	20 ± 0.33^d^
		80:20	40.46 ± 3.92^ab^
		50:50	23.7 ± 1.30^cd^
	H_2_O		32.62 ± 2.13^b^
	Hexane		1.19 ± 0.16^e^
	Ethyl acetate		0.29 ± 0.11^e^

*Note:* Values are expressed as mean ± standard deviation (SD) (*n* = 3). Means with different letters are significantly different at *p* < 0.05.

### 3.2. Determination of Secondary Metabolites Contents

#### 3.2.1. TPC, TFC, and CTC

The results have demonstrated that all berry extracts are rich in phenolic compounds. Samples obtained through PUAE exhibited significantly higher phenolic compound contents compared with those obtained through maceration. This clearly indicates that ultrasonic extraction is generally more efficient than maceration for phenolic compound extraction. However, nonpolar solvents such as ethyl acetate and hexane were less effective for phenolic extraction, particularly under PUAE conditions. Among the extracts, those with the highest TPC were ethanol (26.08 ± 0.22 mgGAE/gDW), aqueous (25.17 ± 0.36 mgGAE/gDW), acetone (24.42 ± 0.30 mgGAE/gDW), and methanol (23.02 ± 0.30 mgGAE/gDW), respectively. Similar findings were observed for TFC and the CTC. Furthermore, Table 2 presents also the extracts with the lowest levels of TPC, which were observed for hexane and ethyl acetate in both extractions, respectively.

**Table 2:** TPC, TFC, and CTC of probe ultrasonic assisted extract and maceration extracts of *Lycium intricatum* berry.

Extraction method	Extraction solvents (solvent:water)		Phytochemical content		
			TPC (mgGAE/gDW)	TFC mgCE/gDW	CTC mgCE/100gDW
Maceration	MeOH	100:0	5.42 ± 0.52^cd^	4.28 ± 0.63^d^	15.71 ± 0.71^d^
		80:20	8.42 ± 0.36^b^	6.06 ± 0.42^cd^	21.14 ± 0.43^cd^
		50:50	7.78 ± 0.50^bc^	5.28 ± 0.10^cd^	18.86 ± 0.14^cd^
	EtOH	100:0	1.00 ± 0.29^d^	1.81 ± 0.08^d^	13.29 ± 0.29^d^
		80:20	2.92 ± 1.45^d^	5.00 ± 0.44^cd^	20.79 ± 0.64^cd^
		50:50	10.22 ± 0.57^ab^	9.28 ± 1.27^bc^	25.33 ± 0.58^c^
	Acetone	100:0	0.75 ± 0.08^d^	1.68 ± 0.85^d^	37.21 ± 0.64^b^
		80:20	11.42 ± 0.17^a^	6.23 ± 0.96^bcd^	42.62 ± 0.82^b^
		50:50	9.42 ± 0.08^abc^	3.11 ± 0.10^d^	30.86 ± 0.29^bc^
	H_2_O		5.22 ± 0.34^cd^	5.22 ± 0.51^cd^	16.71 ± 0.00^d^
	Hexane		3.06 ± 0.05^d^	11.78 ± 0.69^b^	156.00 ± 0.71^a^
	Ethyl acetate		7.53 ± 0.64^bc^	23.72 ± 0.51^a^	157.14 ± 0.71^a^

Ultrasound probe assisted extraction	MeOH	100:0	6.56 ± 0.25^d^	5.39 ± 1.11^c^	134.07 ± 0.64^b^
		80:20	23.02 ± 0.30^ab^	21.78 ± 0.79^a^	165.95 ± 0.36^a^
		50:50	9.72 ± 19^c^	7.33 ± 0.93^c^	157.14 ± 0.71^ab^
	EtOH	100:0	5.28 ± 0.29^d^	4.61 ± 0.79^c^	158.64 ± 0.79^ab^
		80:20	12.22 ± 1.81^c^	13.61 ± 1.11^b^	161.71 ± 1.29^a^
		50:50	26.08 ± 0.22^a^	19.89 ± 0.92^a^	178.33 ± 0.22^a^
	Acetone	100:0	5.06 ± 0.10^d^	6.39 ± 0.51^c^	179.07 ± 0.50^a^
		80:20	24.42 ± 0.30^a^	22.06 ± 1.40^a^	188.62 ± 0.68^a^
		50:50	21.86 ± 0.25^b^	18.00 ± 0.33^ab^	171.14 ± 0.86^a^
	H_2_O		25.17 ± 0.36^a^	22.89 ± 1.23^a^	163.86 ± 0.57^a^
	Hexane		2.89 ± 0.27^d^	3.11 ± 0.63^c^	26.79 ± 0.21^c^
	Ethyl acetate		6.56 ± 0.60^d^	16.94 ± 0.35^ab^	26.71 ± 0.14^c^

*Note:* Values are expressed as mean ± standard deviation (SD) (*n* = 3). Means with different letters are significantly different at *p* < 0.05.

Among the solvent:water mixtures tested (ethanol, methanol, and acetone), those that demonstrated the highest extraction efficiency and resulted in the highest phenolic compound content were as follows: (MeOH:water 80:20), (EtOH:water 50:50), and (acetone:water 80:20). These extracts, including aqueous extracts of PUAE, as well as hexane and ethyl acetate extracts obtained through maceration, were chosen for further analysis in this study.

### 3.3. In Vitro Antioxidant Activity

The *I* *C*_50_ values, representing the concentration of plant extract required to scavenge 50% of the DPPH radicals and hydrogen peroxide, are provided in Table 3. In comparison to ascorbic acid, all extracts demonstrated moderate antioxidant activity, evident in their ability to inhibit DPPH and hydrogen peroxide. The ethanol extract exhibited the most potent antioxidant effect (*I* *C*_50 DPPH_ = 273.90 ± 0.96 *μg*/mL, IC_50 H2O2_ = 30.96 ± 1.95 *μg*/mL), whereas the ethyl acetate extract showed the lowest activity (IC_50 DPPH_ = 301.06 ± 6.33 *μg*/mL, IC_50 H2O2_ = 513.23 ± 4.47 *μg*/mL).

**Table 3:** Scavenging activity of *Lycium intricatum* fruit extracts.

Scavenging activity (*I* *C*_50_ *μ*g/mL)		
	DPPH assay	H_2_O_2_ assay
FM	290.50 ± 6.72^d^	53.19 ± 1.56^c^
FE	273.90 ± 0.96^b^	30.96 ± 1.95^a^
FA	281.36 ± 0.99^c^	61.08 ± 0.82^d^
FAq	295.31 ± 3.79^e^	66.88 ± 1.51^e^
FAE	301.06 ± 6.33^f^	513.23 ± 4.47^g^
FH	282.39 ± 3.72^c^	193.74 ± 3.97^f^
AA	19.90 ± 0.14^a^	35.41 ± 0.23^b^

*Note:* Values are expressed as mean ± standard deviation (SD) (*n* = 3). Means with different letters are significantly different at *p* < 0.05.

Abbreviations: AA, ascorbic acid; FA, acetone extract; FAE, ethyl acetate extract; FAq, aqueous extract; FE, ethanol extract; FH, hexane extract; FM, methanol extract.

### 3.4. In Vitro Antiprotein Denaturation Activity

**Figure 2:**
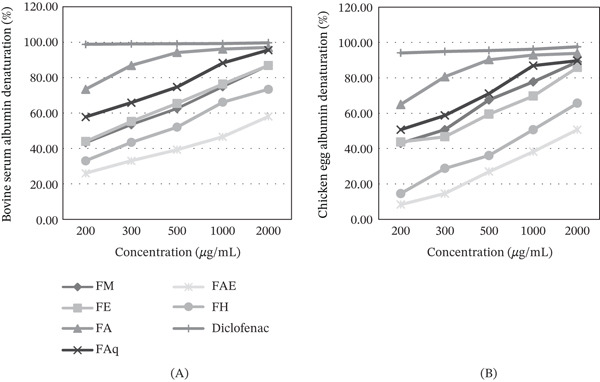
In vitro protein antidenaturation activity of *Lycium intricatum* fruit extracts. (A) Bovine serum albumin denaturation (%); (B) chicken egg albumin denaturation (%); FM, methanol extract; FA, acetone extract; FE, ethanol extract; FAq, aqueous extract; FAE, ethyl acetate extract; FH, hexane extract.

At all concentrations examined, the fruit extracts showcased antiprotein denaturation properties against BSA and CEA. The degree of protein denaturation decreased with increasing extract concentration, indicating a dose‐dependent protective effect. Figure 2 illustrates a significant difference (*p* < 0.05) among the various extracts. The acetone extract demonstrated the highest antiprotein denaturation effect, closely approaching that of the standard. The aqueous, methanolic, and ethanolic extracts were closely followed. On the contrary, the ethyl acetate and hexane extracts exhibited the weakest antiprotein denaturation activity.

### 3.5. Correlation Analysis

**Figure 3:**
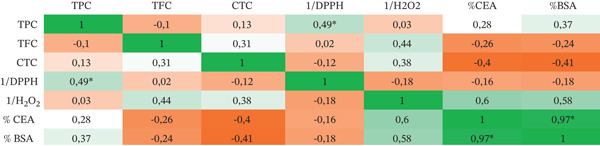
Pearson correlation heat map of secondary metabolite content, antioxidant activity, and antiprotein denaturation activity. *Significant at *p* < 0.05; H_2_O_2_, hydrogen peroxide scavenging activity; DPPH, DPPH scavenging activity; TPC, total phenolic contents; TFC, total flavonoid contents; CTC, condensed tannin content; CEA, chicken egg albumin denaturation; BSA, bovine serum albumin denaturation.

The correlation between TPC, TFC, CTC, antioxidant activity, and antiprotein denaturation activity was assessed using the Pearson coefficient, as illustrated in Figure 3. The coefficient (*r*) was determined to be 0.49, indicating a positive correlation between TPC and DPPH scavenging (*p* < 0.05). On the contrary, a nonsignificant correlation was observed between TFC, CTC, and DPPH. Additionally, hydrogen peroxide inhibition showed a nonsignificant correlation with TPC and TFC content.

No significant correlation was also observed between TPC, TFC, or CTC and the percentage inhibition of egg albumin or BSA. Similarly, the correlations between the DPPH and antidenaturation activity were not significant. However, a moderate positive trend was observed between H₂O₂ and antidenaturation activity (r ≈0.60), suggesting a possible link between hydrogen peroxide scavenging and protein protection. A strong and significant correlation was found between the two antidenaturation assays with *r* = 0.97, *p* < 0.05, confirming the consistency of the results.

### 3.6. HPLC‐UV‐MS/MS Analysis

The HPLC analysis revealed variations in the composition of the extracts depending on the solvent used. Phenolic compounds, including phenolic acids, flavonoids, and carotenoids, were abundant in all extracts. Quercetin and its derivatives were the most prevalent compounds. Two substances, 2 ^′^‐hydroxy‐3,4,4 ^′^,6 ^′^‐tetramethoxychalcone and 5‐O‐Caffeoylquinic acid, were consistently found in all extracts.

**Figure 4:**
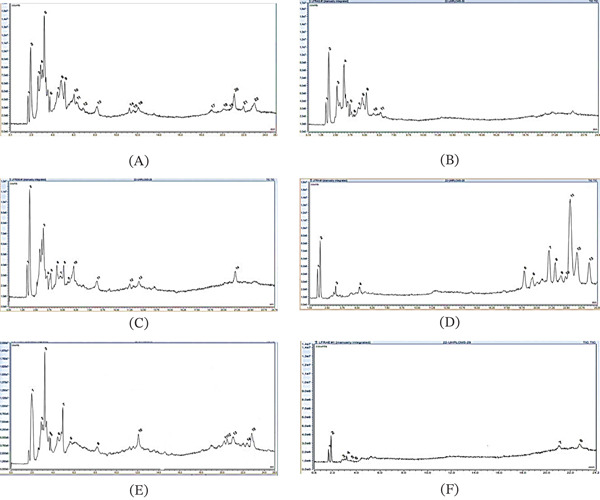
HPLC‐UV‐MS/MS of *Lycium intricatum* fruit extract. (A) HPLC‐UV‐MS/MS of *L. intricatum* fruit extract of methanol; (B) HPLC‐UV‐MS/MS of *L. intricatum* fruit aqueous extract; (C) HPLC‐UV‐MS/MS of *L. intricatum* fruit extract of ethanol; (D) HPLC‐UV‐MS/MS of *L. intricatum* fruit extract of hexane; (E) HPLC‐UV‐MS/MS of *L. intricatum* fruit extract of acetone; (F) HPLC‐UV‐MS/MS of *L. intricatum* fruit extract of ethyl acetate.

The results presented in Table 4 and Figure 4 demonstrated that the ethyl acetate extract contained fewer compounds compared with the other samples, followed by the aqueous extract. The ethanol extract exhibited the presence of 3,5‐Di‐O‐caffeoylquinic acid. Acetone, on the other hand, was effective in extracting myricetin rhamnoside. In contrast, carotenoids (8‐Apo‐*β*‐carotenal and alloxanthin) were detected only in the hexane extract. Overall, phenolic compounds were detected in all extracts, with methanol, ethanol, and acetone proving to be the most efficient solvents for their extraction.

**Table 4:** Phenolic and derivative characterization of *L. intricatum* fruit using HPLC‐UV‐MS/MS.

Tentative identification		*L. intricatum* fruit Extracts						RT	Ref	Chemical formula	Molecular weight (g/mol)	[M‐H]‐ (m/z)	Fragments (*m*/*z*)
		M	E	A	AQ	H	EA						
**Flavonoids**													
**Flavonol**	Quercetin	+	+	+	—	+	+	21.03	S	C_15_H_10_O_7_	302	301	301 271
	*Quercetin 3* ^′^ *-methyl ether*	+	+	+	+	+	—	5.13	N	C_16_H_12_O_7_	316	315	271 255 300
	*Quercetin-3-O-pentoside*	+	—	+	—	+	—	22.01	[23]	C_20_H_18_O_1_	434	433	301 271
	*Quercitrin hydrate*	+	—	+	—	+	+	22.79	[24]	C_21_H_20_O_11_	448	447	301 271
	*Myricetin rhamnoside*	—	—	+	—	—	—	20.47	[25]	C_21_H_20_O_12_	464	463	316 317
	*Isoquercetin*	—	—	—	—	+	—	21.55	N	C_21_H_20_O_12_	464	463	300 301 271
**Chalcone**	*2* ^′^ *-hydroxy-3,4,4* ^′^ *,6* ^′^ *- tetramethoxychalcone*	+	+	+	+	+	+	1.93	N	C_19_H_20_O_6_	344	343	313 315
**Phenolic acid**													
**Hydroxybenzoic acids**	Gallic acid	+	+	—	+	—	—	6.23	N	C_7_H_6_O_5_	170	169	125
**Hydroxycinnamic acid**	5‐*O*‐Caffeoylquinic acid	+	+	+	+	+	+	3.23	[26]	C_16_H_18_O_9_	354	353	191 173
	Coumaroyl 5‐*O*‐caffeoylquinic	+	+	+	+	—	—	4.45	[26]	C_25_H_24_O_11_	500	499	353
	3,4‐Dicaffeoylquinic acid	+	+	+	—	—	—	8.17	[27]	C_25_H_24_O_12_	516	515	353 191
	Chlorogenic acid	+	+	+	—	—	—	12.05	[28]	C_16_H_18_O_9_	354	353	191
	Ferulic acid	+	—	—	—	—	—	18.88	S	C_10_H_10_O_4_	194	193	134 178
	3,5‐Di‐*O*‐caffeoylquinic acid	—	+	—	—	—	—	11.24	N	C_25_H_24_O_12_	516	515	353 179
**Carotenoids**													
**Carotenes**	*8-Apo-β-carotenal*	—	—	—	—	+	—	23.35	N	C_30_H_40_O	416	415	399 293
**Xanthophylls**	*Alloxanthin*	—	—	—	—	+	—	24.37	N	C_40_H_52_O_2_	564	563	563 545

Abbreviations: A, acetone; AQ, aqueous; E, ethanol; EA, ethyl acetate; H, hexane; M, methanol; N, Nist ms/ms library; REF, references; RT, retention time; S, standard.

## 4. Discussion

Several parameters are involved in the choice of extraction solvent: polarity, solubility, selectivity, safety, and so on. [29]. Previous studies have reported notable variations in extraction yields based on the choice of different extraction solvents [30]. Ethanol, methanol, and water are highly polar compared with ethyl acetate, acetone, and hexane. This heightened polarity makes them excellent solvents for dissolving a variety of phenolic compounds. Compared with pure solvents, solvent:water mixtures provided the highest extraction yield. The presence of water, recognized as the most polar solvent, amplifies the polarity of the extraction medium, facilitating the dissolution of a broad spectrum of compounds and substances [29, 31].

Ethanol:water (50:50) and methanol:water (80:20) stand out as superior solvents for phenolic extraction due to their well‐balanced polarity and effective dissolution capabilities for a broad spectrum of phenolic compounds. Ethanol achieves successful dissolution of both polar and nonpolar phenolics with its 50:50 ratio. Conversely, the 80:20 ratio in methanol optimizes solubility while retaining crucial polarity, significantly enhancing the overall efficiency of phenolic compound extraction. Acetone has demonstrated its efficacy in extracting TPC, TFC, and CTC, as previously discussed [22, 32], particularly in the acetone:water mixture (80:20), which proves especially effective. This mixture, enriched with acetone, creates a less polar environment, promoting the solubility of phenolic compounds, particularly those with nonpolar properties.

PUAE has proven to be more effective than maceration. The extraction yields and content of TPC, TFC, and CTC exhibited a notable difference between PUAE and maceration. These findings align with previous studies, highlighting the effectiveness of ultrasonic extraction [33, 34]. Plant cell walls are broken down by the cavitation force produced by ultrasound, which also promotes the effective release of intracellular components. [35]. The main advantages of using PUAE are a reduction in both extraction time and solvent use. However, employing ultrasonic extraction at frequencies exceeding 20 KHz can lead to the generation of free radicals, potentially posing challenges and affecting the integrity of active phytochemicals [36]. In contrast, for nonpolar solvents such as hexane and ethyl acetate, maceration may be more effective than PUAE. Studies have shown that conventional maceration provides prolonged contact time without the potential localized disturbances caused by ultrasound, which may not be optimal in nonpolar systems where cavitation is less effective due to poor transmission of ultrasonic energy in less polar solvents [37]. Polarity, melting and boiling points, density, specific gravity, and affinity are some of the variables that account for the observed differences. In addition, extraction parameters may not be appropriate for the solvent, intermediates and final reaction products, as well as for potential interactions between the solvent and target compounds during the extraction process [38].

In some extracts, particularly FAE, TFC exceeded TPC values. This can be explained by methodological interference. The aluminum chloride method overestimates flavonoid content in the presence of high condensed tannin levels, as tannins also chelate AlCl_3_. Indeed, FAE had the highest CTC. Conversely, the Folin–Ciocalteu method may underestimate phenolics in tannin‐rich samples. HPLC‐MS/MS confirmed low phenolic diversity in FAE despite high TFC/CTC values [39].

In a comparative context, an earlier investigation on Tunisian *L. intricatum* fruit revealed a TPC of (33.60 mg EAG/g DW), marginally surpassing the TPC measured in the Moroccan *L. intricatum* samples tested. Moreover, the TFC registered (12.32 mg EQ/g DW), indicating a slightly lower content compared with the results obtained in this study [11].

The results of antioxidant activity emphasize the potential of aqueous solvent mixtures to yield potent antioxidant effects [22, 40].Variances in antioxidant activity among the extracts can be attributed to their distinct chemical compositions and solubility characteristics, underscoring the importance of selecting suitable solvents and extraction methods to obtain extracts with the intended bioactivities.

The positive correlation observed between TPC and DPPH indicates that the antioxidant effect is directly associated with the content of total phenolic compounds as already reported [16]. However, there has been no observed correlation between TPC, TFC, CTC, and hydrogen peroxide scavenging activity. This suggests that the observed scavenging of hydrogen peroxide may not be directly linked to the phenolic content of the extracts. Other bioactive substances could potentially contribute to the observed antioxidant activity and the absence of clear link emphasizes the complexity of antioxidant action in the fruit extracts [41]. This insignificant correlation can also be explained by the distinct mechanism involved in neutralizing H_2_O_2_, which differs from the direct scavenging of free radicals. Unlike DPPH, hydrogen peroxide is not a radical species, and its scavenging requires decomposition, such as chelation of metal ions or conversion to water and oxygen, instead of a direct donation of hydrogen atoms [42]. These findings show that phenolic compounds′ structure, reactivity, and particular test mechanism all affect antioxidant activity in addition to their overall concentration. Interestingly, CTC exhibited a weak negative correlation with DPPH activity (*r* = −0.1204). These results are consistent with the previous findings [43], who note that the steric accessibility of the DPPH· radical is a determining factor. Condensed tannins are high‐molecular‐weight oligomers that react slowly with DPPH, unlike smaller molecules, which exhibit higher antioxidant capacity. Furthermore, some tannins may exhibit pro‐oxidant behavior or form complexes, further reducing the net scavenging capacity [44].

The analysis of antiprotein denaturation yielded results consistent with those obtained from antioxidant activity assessments. Methanolic, aqueous, and ethanolic extracts demonstrated effective inhibition of protein degradation when compared with control, whereas the strongest antiprotein denaturation effect was observed in acetone extract. Some bioactive compounds responsible for denaturation inhibition may have a greater affinity to acetone and may form stable complexes with proteins, enhancing their ability to prevent denaturation [32].

Among all the extracts, the hexane extract consistently exhibited the lowest protein antidenaturation activity. This can be attributed to its low polarity, which limits the extraction of polar bioactive compounds such as phenolic acids; as shown by the HPLC results; known to play a key role in inhibiting protein denaturation. The presence of the carotenoids, alloxanthin and 8‐apo‐*β*‐carotene in the hexane extract, although interesting from a phytochemical perspective, does not appear to confer strong antiprotein denaturation activity. This observation is consistent with previous reports suggesting that carotenoids offer limited, if any, direct protection against heat‐induced protein denaturation, compared to extracts rich in phenolic acids. Indeed, phenolic compounds and flavonoids show stronger correlations with protein denaturation inhibition than carotenoid*s* [45].

Regarding antiprotein denaturation activity, there were no apparent correlations with TPC, TFC, or CTC. However, moderate negative trends for CTC (r ≈ –0.40) suggest that high tannin levels may reduce protein protection via precipitation as shown by a previous study [46]. A moderate positive trend was also noted between H_2_O_2_ scavenging and antidenaturation (r ≈ 0.60), which suggests that oxidative stress protection may partially contribute to protein stabilization [47].

*L. intricatum* is enriched with bioactive compounds known for their therapeutic and anti‐inflammatory properties [13]. These findings shed light on the possible applications of these extracts in the prevention of protein denaturation‐related disorders and present new prospects for utilizing *L. intricatum* fruit as a potential anti‐inflammatory agent, considering that anti‐inflammatory agents are compounds that inhibit over 20% of protein degradation [48].

Lycium berry has consistently been recognized for its diverse array of phenolic compounds, a fact well‐documented in numerous studies [12, 49]. Depending on the polarity and solubility of the extraction solvent, a range of compounds including flavonoids, phenolic acids, and carotenoids was discerned in the different extracts. The presence of glycosidic flavonoids and hydroxycinnamic acid derivatives stands as a characteristic feature in *Lycium* berry extracts, as elucidated [11, 50]. HPLC‐MS/MS analysis revealed the presence of several phenolic compounds in *L. intricatum* extracts, including quercetin‐diglucoside, caffeic acid, caffeoyl quinic acid, and chlorogenic acid. These qualitative results are generally consistent with the quantitative determination of phenolic content. Indeed, the acetone extract, which exhibited the highest protein antidenaturation activity and the highest polyphenol content, also contained significant levels of quercetin derivatives and chlorogenic acid, compounds known for their strong antioxidant and protein‐stabilizing properties. For the ethyl acetate extract, HPLC‐MS/MS revealed low phenolic diversity, whereas spectrophotometric assays indicated high TFC and CTC values. This apparent contradiction can be explained by the fact that the detection of higher molecular weight proanthocyanidins is restricted in mass spectrometry due to ionization and elution difficulties [51]. 8‐Apo‐*β*‐carotenal and Alloxanthin were not previously identified in *L. intricatum* fruit. These carotenoids are potent antioxidants known for their healing, antiproliferative, anti‐inflammatory, and skin photo protective properties. Their potential use as nutraceutical or cosmetic components is promising for addressing disorders associated with oxidative stress [52].

The antioxidant and anti‐inflammatory characteristics of quercetin, quercetin derivatives [53], phenolic acids, and chlorogenic acid [54] have shown promise in scientific studies and may be beneficial for eye health. Myricetin has been identified as a potential agent for the management of glaucoma [55]. Furthermore, gallic acid has demonstrated potential in preventing corneal staining caused by sodium fluorescein and inhibiting apoptosis of corneal epithelial cells [56]. And by regulating inflammation and preserving the ocular surface, dicaffeoylquinic acid has been shown to improve dry eyes [57]. This may explain the traditional use of this fruit by the Moroccan population to treat eye problems. Other studies, however, are required in order to confirm their efficacy in treating certain eye diseases.

## 5. Conclusion

The extraction solvent and method significantly influence the recovery of bioactive compounds from *L. intricatum* fruit, with solvent‐water mixtures proving to be the most effective. *L. intricatum* fruit exhibited the highest antiprotein denaturation activity, mainly associated with acetone extracts rich in quercetin derivatives, highlighting its potential as a natural agent against protein aggregation and inflammation. Whereas the hexane extract showed the lowest. Although TPC correlated positively with DPPH activity, no such correlation was observed with H_2_O_2_ scavenging or antidenaturation, highlighting the complexity of the antioxidant mechanisms. Importantly, HPLC‐MS/MS identified alloxanthin and 8‐apo‐*β*‐carotene for the first time in this species and have been detected in the hexane extract. Phenolic acid and chlorogenic acid, recognized for their therapeutic potential in addressing ocular issues, are also identified. These findings shed light on the traditional use of *L. intricatum* fruits for ocular treatments and highlight their richness in bioactive compounds with promising therapeutic potential. Further studies are warranted to explore their pharmaceutical and medical applications.

## Funding

No funding was received for this manuscript.

## Conflicts of Interest

The authors declare no conflicts of interest.

## Data Availability

The data used to support the results of this study are included in the article. However, additional information may be obtained from the corresponding author upon request.

## References

[1] Hao D. C. , Hao D. C. , Chapter 1—Genomics and Evolution of Medicinal Plants, Ranunculales Medicinal Plants, 2019, Academic Press, 1–33, 10.1016/B978-0-12-814232-5.00001-0.

[2] Petrovska B. B. , Historical Review of Medicinal Plants′ Usage, Pharmacognosy Reviews. (2012) 6, no. 11, 1–5, 10.4103/0973-7847.95849, 2-s2.0-84862061215, 22654398. 22654398 PMC3358962

[3] Bellakhdar J. , Contribution à l’étude de la pharmacopée traditionnelle au Maroc: la situation actuelle, les produits, les sources du savoir (enquête ethnopharmacologique de terrain réalisée de 1969 à 1992) [Thèse de doctorat], 1997, Université de Metz.

[4] Bellakhdar J. , A New Look at Traditional Medicine in Morocco, World Health Forum. (1989) 10, no. 2, 193–199, 2610831. 2610831

[5] Kachmar M. R. , Naceiri Mrabti H. , Bellahmar M. , Ouahbi A. , Haloui Z. , El Badaoui K. , Bouyahya A. , and Chakir S. , Traditional Knowledge of Medicinal Plants Used in the Northeastern Part of Morocco, Evidence-Based Complementary and Alternative Medicine. (2021) 2021, no. 2021, e6002949, 10.1155/2021/6002949, 34512779. PMC8426073 34512779

[6] Zheng X. H. , Huang Y. P. , Liang Q. P. , Xu W. , Lan T. , and Zhou G. X. , A New Lignanamide From the Root of *Lycium yunnanense* Kuang and Its Antioxidant Activity, Molecular Journal of Synthetic Chemistry and Natural Products Chemistry. (2018) 23, no. 4, 10.3390/molecules23040770, 2-s2.0-85044751207, 29584684. PMC6017700 29584684

[7] Nogales M. , Delgado J. D. , and Medina F. M. , Shrikes, Lizards and *Lycium intricatum* (Solanaceae) Fruits: A Case of Indirect Seed Dispersal on an Oceanic Island (Alegranza, Canary Islands), Journal of Ecology. (1998) 86, no. 5, 866–871, 10.1046/j.1365-2745.1998.8650866.x, 2-s2.0-0031768688.

[8] Yao R. , Heinrich M. , and Weckerle C. S. , The Genus *Lycium* as food and Medicine: A Botanical, Ethnobotanical and Historical Review, Journal of Ethnopharmacology. (2018) 15, no. 212, 50–66, 10.1016/j.jep.2017.10.010, 2-s2.0-85032171984. 29042287

[9] Bellakhdar J. , Claisse R. , Fleurentin J. , and Younos C. , Repertory of Standard Herbal Drugs in the Moroccan Pharmacopoea, Journal of Ethnopharmacology. (1991) 35, no. 2, 123–143, 10.1016/0378-8741(91)90064-K, 2-s2.0-0026055280. 1809818

[10] Swallah M. S. , Sun H. , Affoh R. , Fu H. , and Yu H. , Antioxidant Potential Overviews of Secondary Metabolites (Polyphenols) in Fruits, International Journal of Food Science. (2020) 2020, no. 1, 9081686, 10.1155/2020/9081686. 32455130 PMC7229537

[11] Abdennacer B. , Karim M. , Yassine M. , Nesrine R. , Mouna D. , and Mohamed B. , Determination of Phytochemicals and Antioxidant Activity of Methanol Extracts Obtained From the Fruit and Leaves of Tunisian *Lycium intricatum* Boiss, Food Chemistry. (2015) 174, no. 174, 577–584, 10.1016/j.foodchem.2014.11.114, 2-s2.0-84914125489, 25529722. 25529722

[12] Bendjedou H. , Maggi F. , Bennaceur M. , Mancinelli M. , Benamar H. , and Barboni L. , *A New Ionone Derivative From Lycium intricatum* Boiss. (Solanaceae), Natural Product Research. (2022) 36, no. 3, 687–694, 10.1080/14786419.2020.1797729. 32705905

[13] Boulila A. and Bejaoui A. , *Lycium intricatum* Boiss.: An Unexploited and Rich Source of Unsaturated Fatty Acids, 4-Desmethylsterols and Other Valuable Phytochemicals, Lipids in Health and Disease. (2015) 24, no. 14, 10.1186/s12944-015-0055-9, 2-s2.0-84933531331. PMC4485344 26104186

[14] Yu J. , Yan Y. , Zhang L. , Mi J. , Yu L. , Zhang F. , Lu L. , Luo Q. , Li X. , Zhou X. , and Cao Y. , A Comprehensive Review of Goji Berry Processing and Utilization [Internet], 11, no. 12, 7445–7457, 10.1002/fsn3.3677, 38107149. PMC10724590 38107149

[15] Zhong Y. , Shahidi F. , and Naczk M. , Phytochemicals and Health Benefits of Goji Berries, Dried Fruits, 2013, Academic Press, 10.1002/9781118464663.ch6, 2-s2.0-84887362916.

[16] Bouaouda K. , Elagdi C. , El Hachlafi N. , Mohtadi K. , Hsaine M. , Kettani A. , Flouchi R. , Goh K. W. , Bouyahya A. , Mrabti H. N. , Saile R. , and Taki H. , HPLC-UV-MS/MS Profiling of Phenolics From *Euphorbia nicaeensis* (All.) Leaf and Stem and Its Antioxidant and Anti-Protein Denaturation Activities, Progress In Microbes & Molecular Biology. (2023) 6, no. 1, 10.36877/pmmb.a0000331.

[17] Herrero M. , Temirzoda T. N. , Segura-Carretero A. , Quirantes R. , Plaza M. , and Ibañez E. , New Possibilities for the Valorization of Olive Oil By-Products, Journal of Chromatography A. (2011) 1218, no. 42, 7511–7520, 10.1016/j.chroma.2011.04.053, 2-s2.0-80053305619. 21600577

[18] Barros L. , Cabrita L. , Boas M. V. , Carvalho A. M. , and Ferreira I. C. F. R. , Chemical, Biochemical and Electrochemical Assays to Evaluate Phytochemicals and Antioxidant Activity of Wild Plants, Food Chemistry. (2011) 127, no. 4, 1600–1608, 10.1016/j.foodchem.2011.02.024, 2-s2.0-79953182002.

[19] Salar R. K. and Purewal S. S. , Improvement of DNA Damage Protection and Antioxidant Activity of Biotransformed Pearl Millet (*Pennisetum glaucum*) Cultivar PUSA-415 using *Aspergillus oryzae* MTCC 3107, Biocatalysis and Agricultural Biotechnology. (2016) 1, no. 8, 221–227, 10.1016/j.bcab.2016.10.005, 2-s2.0-84991666622.

[20] Qamar M. , Akhtar S. , Ismail T. , Yuan Y. , Ahmad N. , Tawab A. , Ismail A. , Barnard R. T. , Cooper M. A. , Blaskovich M. A. T. , and Ziora Z. M. , *Syzygium cumini* (L.),Skeels Fruit Extracts: In Vitro and in Vivo Anti-Inflammatory Properties, Journal of Ethnopharmacology. (2021) 271, no. 271, 113805, 10.1016/j.jep.2021.113805, 33465442. 33465442

[21] Saravanan M. , Senthilkumar P. , Chinnadurai V. , Murugesan Sakthivel K. , Rajeshkumar R. , and Pugazhendhi A. , Antiangiogenic, Anti-Inflammatory and Their Antioxidant Activities of *Turnera subulata* Sm. (Turneraceae), Process Biochemistry. (2020) 1, no. 89, 71–80, 10.1016/j.procbio.2019.10.011.

[22] Sultana B. , Anwar F. , and Ashraf M. , Effect of Extraction Solvent/Technique on the Antioxidant Activity of Selected Medicinal Plant Extracts, Molecules. (2009) 14, no. 6, 2167–2180, 10.3390/molecules14062167, 2-s2.0-67649836502, 19553890. 19553890 PMC6254218

[23] Li C. and Seeram N. P. , Ultra-Fast Liquid Chromatography Coupled With Electrospray Ionization Time‐of‐Flight Mass Spectrometry for the Rapid Phenolic Profiling of Red Maple (*Acer rubrum*) leaves, Journal of Separation Science. (2018) 41, no. 11, 2331–2346, 10.1002/jssc.201800037, 2-s2.0-85044846977, 29512337. 29512337 PMC7167591

[24] Puigventós L. , Navarro M. , Alechaga É. , Núñez O. , Saurina J. , Hernández-Cassou S. , and Puignou L. , Determination of Polyphenolic Profiles by Liquid Chromatography-Electrospray-Tandem Mass Spectrometry for the Authentication of Fruit Extracts, Analytical and Bioanalytical Chemistry. (2015) 407, no. 2, 597–608, 10.1007/s00216-014-8298-2, 2-s2.0-84937913636, 25370163. 25370163

[25] Bystrom L. M. , Lewis B. A. , Brown D. L. , Rodriguez E. , and Obendorf R. L. , Characterization of Phenolics by LC-UV/vis, LC-MS/MS and Sugars by GC in *Melicoccus bijugatus* Jacq. ‘Montgomery’ fruits, Food Chemistry. (2008) 111, no. 4, 1017–1024, 10.1016/j.foodchem.2008.04.058, 2-s2.0-46749119759. 21709744 PMC3123376

[26] Gouveia S. C. and Castilho P. C. , Characterization of Phenolic Compounds in *Helichrysum melaleucum* by High-Performance Liquid Chromatography With on-Line Ultraviolet and Mass Spectrometry Detection, Rapid Communications in Mass Spectrometry. (2010) 24, no. 13, 1851–1868, 10.1002/rcm.4585, 2-s2.0-77954247740. 20533315

[27] Zhang Y. , Xiong H. , Xu X. , Xue X. , Liu M. , Xu S. , Liu H. , Gao Y. , Zhang H. , and Li X. , Compounds Identification in Semen Cuscutae by Ultra-High-Performance Liquid Chromatography (UPLCs) Coupled to Electrospray Ionization Mass Spectrometry, Molecules. (2018) 23, no. 5, 10.3390/molecules23051199, 2-s2.0-85047181551. PMC6100538 29772791

[28] Grati W. , Samet S. , Bouzayani B. , Ayachi A. , Treilhou M. , Téné N. , and Mezghani-Jarraya R. , HESI-MS/MS Analysis of Phenolic Compounds From *Calendula aegyptiaca* Fruits Extracts and Evaluation of Their Antioxidant Activities, Molecules. (2022) 27, no. 7, 10.3390/molecules27072314, 35408713. PMC9000822 35408713

[29] Abubakar A. R. and Haque M. , Preparation of Medicinal Plants: Basic Extraction and Fractionation Procedures for Experimental Purposes, Journal of Pharmacy and Bioallied Sciences. (2020) 12, no. 1, 1–10, 10.4103/jpbs.JPBS_175_19. 32801594 PMC7398001

[30] Ngo T. V. , Scarlett C. J. , Bowyer M. C. , Ngo P. D. , and Vuong Q. V. , Impact of Different Extraction Solvents on Bioactive Compounds and Antioxidant Capacity From the Root of *Salacia chinensis* L, Journal of Food Quality. (2017) 2017, no. 2017, e9305047, 10.1155/2017/9305047, 2-s2.0-85013419010.

[31] Boeing J. S. , Barizão E. O. , E Silva B. C. , Montanher P. F. , de Cinque Almeida V. , and Visentainer J. V. , Evaluation of Solvent Effect on the Extraction of Phenolic Compounds and Antioxidant Capacities From the Berries: Application of Principal Component Analysis, Chemistry Central Journal. (2014) 8, no. 1, 10.1186/s13065-014-0048-1, 2-s2.0-84910097458, 25246942. PMC4158270 25246942

[32] Zuorro A. , Iannone A. , and Lavecchia R. , Water–Organic Solvent Extraction of Phenolic Antioxidants From Brewers′ Spent Grain, Processes. (2019) 7, no. 3, 10.3390/pr7030126, 2-s2.0-85062921992.

[33] Tran C. H. , Nghiem M. T. , Dinh A. M. T. , Dang T. T. N. , Van Do T. T. , Chu T. N. , Mai T. H. , and Phan V. M. , Optimization Conditions of Ultrasound-Assisted Extraction for Phenolic Compounds and Antioxidant Activity from *Rubus alceifolius* Poir Leaves, International Journal of Food Science. (2023) 2023, 10.1155/2023/7576179, 7576179, 37854461. 37854461 PMC10581860

[34] Mahmoud M. H. , Abu-Salem F. M. , and Azab D. E. S. H. , A Comparative Study of Pectin Green Extraction Methods From Apple Waste: Characterization and Functional Properties, International Journal of Food Science. (2022) 2022, no. 1, 2865921, 10.1155/2022/2865921. 36578434 PMC9792233

[35] Picó Y. , Ultrasound-Assisted Extraction for Food and Environmental Samples, TrAC Trends in Analytical Chemistry. (2013) 43, 84–99, 10.1016/j.trac.2012.12.005, 2-s2.0-84873466480.

[36] Nn A. , A Review on the Extraction Methods Use in Medicinal Plants, Principle, Strength and Limitation, Medicinal & Aromatic Plants. (2015) 4, https://www.semanticscholar.org/paper/A-Review-on-the-Extraction-Methods-Use-in-Medicinal-Nn/1f57ac7b5510db3fbf1e5066579e35f2dfdb0b11.

[37] Herrera-Pool E. , Ramos-Díaz A. L. , Lizardi-Jiménez M. A. , Pech-Cohuo S. , Ayora-Talavera T. , Cuevas-Bernardino J. C. , García-Cruz U. , and Pacheco N. , Effect of Solvent Polarity on the Ultrasound Assisted Extraction and Antioxidant Activity of Phenolic Compounds From Habanero Pepper Leaves (*Capsicum chinense*) and its identification by UPLC-PDA-ESI-MS/MS, Ultrasonics Sonochemistry. (2021) 76, no. 76, 105658, 10.1016/j.ultsonch.2021.105658, 34242865. 34242865 PMC8273200

[38] Dzah C. S. , Duan Y. , Zhang H. , Wen C. , Zhang J. , Chen G. , and Ma H. , The Effects of Ultrasound Assisted Extraction on Yield, Antioxidant, Anticancer and Antimicrobial Activity of Polyphenol Extracts: A Review, Food Bioscience. (2020) 35, no. 35, 100547, 10.1016/j.fbio.2020.100547.

[39] Nastasi J. R. , Colourimetric Assays for Assessing Polyphenolic Phytonutrients with Nutraceutical Applications: History, Guidelines, Mechanisms, and Critical Evaluation, Nutraceuticals. (2025) 5, no. 4, 10.3390/nutraceuticals5040040.

[40] Nakilcioğlu-Taş E. and Ötleş S. , Influence of Extraction Solvents on the Polyphenol Contents, Compositions, and Antioxidant Capacities of Fig (*Ficus carica* L.) Seeds, Anais da Academia Brasileira de Ciências. (2021) 93, no. 1, e20190526, 10.1590/0001-3765202120190526, 33886699. 33886699

[41] Nur Syukriah A. R. , Liza M. S. , Harisun Y. , and Fadzillah A. A. M. , Effect of Solvent Extraction on Antioxidant and Antibacterial Activities From *Quercus infectori*a (Manjakani), International Food Research Journal. (2014) 21, http://mymedr.afpm.org.my/publications/46579.

[42] Pisoschi A. M. and Pop A. , The Role of Antioxidants in the Chemistry of Oxidative Stress: A Review, European Journal of Medicinal Chemistry. (2015) 97, no. 97, 55–74, 10.1016/j.ejmech.2015.04.040, 2-s2.0-84929152649. 25942353

[43] Gulcin İ. , Antioxidants: A Comprehensive Review, Archives of Toxicology. (2025) 99, no. 5, 1893–1997, 10.1007/s00204-025-03997-2. 40232392 PMC12085410

[44] Blažková M. , Uváčková Ľ. , Maliarová M. , Sokol J. , Viskupičová J. , and Maliar T. , Concurrent Analysis of Antioxidant and Pro-Oxidant Activities in Compounds From Plant Cell Cultures, BioTech. (2025) 14, no. 4, 10.3390/biotech14040091. PMC12641827 41283326

[45] Gunathilake K. D. P. P. , Ranaweera K. K. D. S. , and Rupasinghe H. P. V. , In Vitro Anti-Inflammatory Properties of Selected Green Leafy Vegetables, Biomedicines. (2018) 6, no. 4, 10.3390/biomedicines6040107, 2-s2.0-85057597089. PMC6316011 30463216

[46] Kilmister R. L. , Faulkner P. , Downey M. O. , Darby S. J. , and Falconer R. J. , The Complexity of Condensed Tannin Binding to Bovine Serum Albumin—AN isothermal Titration Calorimetry Study, Food Chemistry. (2016) 1, no. 190, 173–178, 10.1016/j.foodchem.2015.04.144, 2-s2.0-84930227021. 26212957

[47] Duong L. D. , West J. D. , and Morano K. A. , Redox Regulation of Proteostasis, Journal of Biological Chemistry. (2024) 300, 107977, 10.1016/j.jbc.2024.107977. 39522946 PMC11664415

[48] Gondkar A. S. , Deshmukh V. K. , and Chaudhari S. R. , Synthesis, Characterization and In-Vitro Anti-Inflammatory Activity of Some Substituted 1,2,3,4 Tetrahydropyrimidine Derivatives, Drug Invention Today. (2013) 5, no. 3, 175–181, 10.1016/j.dit.2013.04.004, 2-s2.0-84884418170.

[49] Jiang Y. , Fang Z. , Leonard W. , and Zhang P. , Phenolic Compounds in *Lycium* Berry: COMPOSITION, Health Benefits and Industrial Applications, Journal of Functional Foods. (2021) 1, no. 77, 104340, 10.1016/j.jff.2020.104340.

[50] Wu W. B. , Hung D. K. , Chang F. W. , Ong E. T. , and Chen B. H. , Anti-Inflammatory and Anti-Angiogenic Effects of Flavonoids Isolated From *Lycium barbarum* Linnaeus on Human Umbilical Vein Endothelial Cells, Food & Function. (2012) 3, no. 10, 1068–1081, 10.1039/C2FO30051F, 2-s2.0-84867013363. 22751795

[51] Reeves S. G. , Condensed Tannin Characterization With FT-ICR MALDI Mass Spectrometry and Separation With Saw-Tooth Gradient HPLC, 2020, Miami University, https://etd.ohiolink.edu/acprod/odb_etd/etd/r/1501/10?clear=10%26p10_accession_num=miami1591185101154831.

[52] Galasso C. , Corinaldesi C. , and Sansone C. , Carotenoids From Marine Organisms: Biological Functions and Industrial Applications, Antioxidants. (2017) 6, no. 4, 10.3390/antiox6040096, 2-s2.0-85036532446, 29168774. PMC5745506 29168774

[53] Sato S. and Mukai Y. , Modulation of Chronic Inflammation by Quercetin: The Beneficial Effects on Obesity, Journal of Inflammation Research. (2020) 4, no. 13, 421–431, 10.2147/JIR.S228361. PMC7425105 32848440

[54] Shahidi F. and Yeo J. , Bioactivities of Phenolics by Focusing on Suppression of Chronic Diseases: A Review, International Journal of Molecular Sciences. (2018) 19, no. 6, 10.3390/ijms19061573, 2-s2.0-85047758559. PMC6032343 29799460

[55] Yang Q. , Li Y. , and Luo L. , Effect of Myricetin on Primary Open-Angle Glaucoma, Translational Neuroscience. (2018) 12, no. 9, 132–141, 10.1515/tnsci-2018-0020, 2-s2.0-85056874092. PMC6234474 30473883

[56] Li K. , Gong Q. , Lu B. , Huang K. , Tong Y. , Mutsvene T. E. , Lin M. , Xu Z. , Lu F. , Li X. , and Hu L. , Anti-Inflammatory and Antioxidative Effects of Gallic Acid on Experimental Dry Eye: In Vitro and In Vivo Studies, Eye and Vision. (2023) 10, no. 1, 10.1186/s40662-023-00334-5, 37122017. PMC10150500 37122017

[57] Yoon C. H. , Jang H. J. , Ryu J. S. , Ko J. H. , Ahn K. S. , Oh S. R. , Oh J. H. , Chung J. H. , and Oh J. Y. , 1,5-Dicaffeoylquinic acid From Pseudognaphalium Affine Ameliorates Dry Eye Disease via Suppression of Inflammation and protection of the Ocular Surface, Ocular Surface. (2023) 29, no. 29, 469–479, 10.1016/j.jtos.2023.06.016, 37390940. 37390940

